# Associations Between Sedentary Behaviour and Fine and Gross Motor Skills in 3‐ to 4‐Year‐Olds: A Secondary Data Analysis From Sunrise International Study Pilot Studies

**DOI:** 10.1111/cch.70092

**Published:** 2025-05-12

**Authors:** Nana A. Kwofie, Adang Suherman, Alex A. Florindo, Amanda Staiano, Amy S. HA, Anthony D. Okely, Asmaa El Hamdouchi, Bang Nguyen Pham, Catherine E. Draper, Chiaki Tanaka, Denise Koh, Dong Hoon Kim, E. Kipling Webster, Hongyan Guan, Hong Kim Tang, John J. Reilly, Kar Hau Chong, Marie Löf, Mark S. Tremblay, Mohammad Sorowar Hossain, Nyaradzai Munambah, Penny L. Cross, Pujitha Wickramasinghe, Thanh Van Kim, Xanne Janssen

**Affiliations:** ^1^ Department of Psychological Sciences and Health University of Strathclyde Glasgow UK; ^2^ Faculty of Sport and Health Education Universitas Pendidikan Indonesia Bandung Indonesia; ^3^ Universidade de Sao Paulo Escola de Artes Ciencias e Humanidades Sao Paulo Brazil; ^4^ Pennington Biomedical Research Center Baton Rouge Louisiana USA; ^5^ Department of Sports Science and Physical Education, Faculty of Education Chinese University of Hong Kong Hong Kong; ^6^ Early Start, School of Health and Society, Faculty of the Arts, Social Sciences and Humanities University of Wollongong Wollongong Australia; ^7^ Unité Mixte de Recherche Nutrition et Alimentation Regional Designated Center of Nutrition Kenitra Morocco; ^8^ Population Health and Demography Unit PNG Institute of Medical Research Goroka Papua New Guinea; ^9^ MRC‐Wits DPHRU University of the Witwatersrand Johannesburg South Africa; ^10^ College of Health and Welfare J F Oberlin University Machida Japan; ^11^ Centre of Community Education and Well‐Being, Faculty of Education Universiti Kebangsaan Bangi Malaysia; ^12^ Korea Institute of Child Care and Education Seoul Republic of Korea; ^13^ Institute of Public and Preventive Health Augusta University Augusta Georgia USA; ^14^ Nurturing Care Research and Guidance Center for Infant and Young Children, Beijing Municipal Key Laboratory of Child Development and Nutriomics Capital Institute of Pediatrics Beijing China; ^15^ Pham Ngoc Thach University of Medicine Ho Chi Minh City Vietnam; ^16^ Department Department of Medicine Huddinge Karolinska Institute Stockholm Sweden; ^17^ Children's Hospital of Eastern Ontario Research Institute Ottawa Ontario Canada; ^18^ Biomedical Research Foundation Dhaka Bangladesh; ^19^ Rehabilitation Sciences Unit, Faculty of Medicine and Health Sciences University of Zimbabwe Harare Zimbabwe; ^20^ Research Services Office, Research and Innovation Division University of Wollongong Wollongong New South Wales Australia; ^21^ Faculty of Medicine University of Colombo Colombo Sri Lanka; ^22^ Department of Epidemiology, Faculty of Public Health Pham Ngoc Thach University of Medicine Ho Chi Minh City Vietnam

**Keywords:** motor competence, motor skills, preschoolers, restrained sitting, screen time

## Abstract

**Background:**

The evidence on associations between sedentary behaviour (SB) and motor skills in 3‐ to 4‐year‐olds is unclear and mostly from high‐income countries.

**Objective:**

The objective of this study is to examine associations between (1) screen time (h/day) and total daily SB (h/day), and gross and fine motor skills, and (2) meeting the restraint, screen time and overall SB (restraint and screen time) guidelines and fine and gross motor skills.

**Methods:**

Cross‐sectional study of 1394 3‐ to 4‐year‐olds from the pilot phase of the SUNRISE International study. Time spent in SB was measured using the *activ*PAL accelerometer, whereas screen time and restraint were measured using a parent questionnaire. Fine and gross motor skills were measured using parent‐reported Ages and Stages Questionnaire (ASQ‐3). Associations between SB and motor skills were determined using linear and logistic regression, adjusting for sex and socioeconomic status.

**Results:**

Every additional 1 h of screen time was associated with 0.50‐point reduction in gross motor skills scores (*p* = 0.008). More screen time was associated with decreased odds of being on track for fine and gross motor skill development (*p* < 0.001 and *p* = 0.017, respectively). Meeting the screen time (*p* = 0.009) and overall SB guidelines (*p* = 0.006) were favourably associated with fine motor skills scores. Meeting the screen time, restraint and overall SB guidelines were favourably associated with gross motor skills scores (*p* = 0.007, *p* < 0.001 and *p* < 0.001, respectively), higher odds of being on track for fine (*p* = 0.033, *p* = 0.015 and *p* < 0.001, respectively) and gross motor skills (*p* = 0.006, *p* < 0.001 and *p* < 0.001, respectively) development.

**Conclusion:**

The present study adds to the evidence on the importance of sedentary behaviour for the motor development of 3‐ to 4‐year‐olds. It is important that policy makers and health educators develop strategies that will encourage and promote adherence to sedentary behaviour guidelines among 3‐ and 4‐year‐olds.

**Summary**

- The WHO recommends that children aged 3–4 years do not accumulate more than 1 h of screen time per day and are not restrained sedentary for more than 1 h at a time.
- Studies suggest that children 3–4 years may be exceeding the WHO sedentary behaviour guideline.
- Findings from this study suggests that children 3–4 years exceeding the guideline recommendation are more likely to suffer from poorer fine and gross motor development.
- Strategies and programmes from policy makers, health educators and other stakeholders may be beneficial to promote and encourage adherence to the SB guidelines.

## 1 Introduction

In 2019, the World Health Organization (WHO) developed the first global guidelines for 24‐h movement behaviours for the under 5 s, which included recommendations for sedentary behaviour (SB) (WHO 2019). The WHO recommends that children aged 3–4 years do not accumulate more than 1 h of screen time per day and are not restrained sedentary for more than 1 h at a time (WHO 2019). Several studies have examined the prevalence of meeting the guidelines, with a wide range of estimates (Kracht, Webster, and Staiano 2020; McArthur et al. 2022; Tanaka et al. 2020; Hinkley et al. 2020; Nyström et al. 2020).

Children's gross motor skills, such as locomotor, object manipulation and stability skills, are instrumental for physical activities and are associated with healthy weight and cardiorespiratory fitness (Henrique et al. 2016; Lima et al. 2017; Cattuzzo et al. 2016). Gross motor skills are also considered the building blocks of movement (Stodden et al. 2008 or Clark and Metcalfe 2002), and children with higher levels of gross motor skills have been shown to spend less time sitting and more time in moderate‐ to vigorous‐intensity physical activities (Webster, Martin, and Staiano 2019). Fine motor skills, such as grasping, object manipulation or drawing, facilitate participation in learning in preschool and early school years (Marr et al. 2003) and are linked positively to cognitive development (Roebers et al. 2014) and academic skills (Cameron et al. 2012).

Two systematic reviews (dos Santos et al. 2021; Kwofie et al. 2024) have examined relationships between SB and motor skills. In addition, a systematic review by Li et al. (2020) and several more recent studies (e.g., Fadhli et al. 2022; Rogović, Šalaj, and Puharić 2022; Kim et al. 2022; Byambaa et al. 2024) have examined the relationship between screen time and motor development, in preschoolers. The results of these systematic reviews and recent studies show inconclusive evidence on the relationship between SB and/or screen time and motor development in young children. In addition, most of these studies were conducted in high‐income countries, and some studies included ‘preschool’ participants up to 6 years old (dos Santos et al. 2021; Li et al. 2020; Rogović, Šalaj, and Puharić 2022), whereas the WHO 2019 Guidelines were developed specifically for children aged up to 4.9 years. Development in children differs for each milestone, and in older children, SB may not have such a significant impact on motor development as in younger children. The 3‐ to 4‐year age group is an important time when specific movement patterns and skills develop, providing the basis for acquisition of future complex skills (Malina 1991). To better understand how SB relates to motor skill development, a focus on 3‐ to 4‐year‐olds will be helpful, as they are distinct from older and younger children. Further investigation with geographically and culturally diverse groups will help understand associations between components of SB (screen time, restraint and total daily accelerometer‐measured SB) and motor skills in 3‐ to 4‐year‐olds.

The aim of the present study was to test for associations between SB and fine and gross motor skills in a diverse international sample of 3‐ to 4‐year‐olds. Specifically, to examine associations between (1) screen time (h/day) and total daily accelerometery measured SB (h/day), and gross and fine motor skills, and (2) meeting the restraint, screen time and overall SB (restraint and screen time combined) guidelines and fine and gross motor skills.

## 2 Methodology

### 2.1 Sampling and Recruitment

Data from all eligible high‐income countries (HICs) and low‐ and middle‐income countries (LMICs) involved in the SUNRISE pilot study were pooled for use in this present study (*n* = 19 countries; see supplementary file 1). Countries were eligible and included if they had collected data on the exposure and outcome measures for the present study.

Participants were recruited using a convenience cluster sampling approach either through Early Childhood Education and Care (ECEC) services or from the community (where ECEC services were limited). In countries where children were exclusively recruited from both public and private ECEC centres, a maximum of 20 children per centre were recruited to reduce the impact of clustering of data (i.e., influence of the centre) within the sample. Participating countries were encouraged to recruit a sample that was stratified in terms of sex, socioeconomic backgrounds of parents and urban/rural residence. All data collectors underwent extensive training to assist with quality assurance and data standardization (Okely et al. 2021).

Ethical approval was received from the University of Wollongong Human Research Ethics Committee (ref. no. 2018/044) and from the local ethics committee(s) in each participating country prior to the study commencing and parents of participants provided written consent before participating.

### 2.2 Participants

Children eligible to participate were boys and girls between the ages of 3.0 and 4.9 years who were able to participate in ambulatory behaviours and could wear an accelerometer (Okely et al. 2021). Children were excluded if they had a physical disability that would preclude a child to engage in physical activity that may be recorded by the accelerometer placed on the thigh. Also, children who did not assent to participation in the study measures were withdrawn from the study.

### 2.3 Measures

An overview of all outcome and exposure measures is shown in Tables 1a and 1b and described in more detail below.

**TABLE 1a:** Continuous variables and measurement tools with their validity and reliability.

Exposure variable	Measurement	Validity and reliability
Screen time (min/day)	Subjective measure using SUNRISE parent questionnaire.	Not validated but practical and culturally appropriate with some face validity (Kariippanon et al. 2022; Hossain et al. 2021)
Restraint (mins/day)	Subjective measure using SUNRISE parent questionnaire.	Not validated but practical and culturally appropriate with some face validity (Kariippanon et al. 2022; Hossain et al. 2021)
Total daily SB (hours/day)	Objective measure using *activ*PAL 4.	Validated and reliable in children under 5 years old (Davies et al. 2012; Davies, Reilly, and Paton 2012; Janssen et al. 2014)

Outcome variable	Measurement	Validity and reliability
Fine motor skills scores (continuous data)	Objective measure using Ages and Stages Questionnaire 3.	Validated in children under 5 years (e.g., Fuengfoo et al. 2020; Shariatpanahi et al. 2024)
Gross motor skills scores (continuous data)	Objective measure using Ages and Stages Questionnaire 3.	Validated in children under 5 years (e.g., Fuengfoo et al. 2020; Shariatpanahi et al. 2024)

**TABLE 1b:** Categorical variable table and measured outcome.

Exposure variable	Measured exposure	Categorized outcome
Screen time guideline	Meeting ≤ 1 h/day of screen time	0—If they did not meet the screen time guideline. 1—if they met the screen time guideline.
Restraint guideline	Meeting ≤ 1 h at a time spent restrained	0—If they did not meet the restraint guideline. 1—if they met the restraint guideline
Overall SB guideline	Meeting ≤ 1 h/day of screen time and ≤ 1 h at a time spent restrained	0—If they did not meet both guidelines. 1—if they met both the guidelines

Outcome variable	Measured outcome	Categorized outcome
Fine motor skills	Total score of the sum of all task score (0, *not yet*; 5, *sometimes*; 10, *yes*)	On track (if total score is above 30.58). At risk (if total score is between 15.81 and 30.58). Delayed (if total score is less than 15.81)
Gross motor skill	Total score of the sum of all task score (0, *not yet*; 5, *sometimes*; 10, *yes*)	On track (if total score is above 42.74). At risk (if total score is between 32.78 and 42.74). Delayed (if total score is less than 32.78)

#### 2.3.1 Sedentary Behaviour

##### 2.3.1.1 Total Daily Accelerometer Measured SB

Total daily SB was measured using the *activ*PAL 4 (PAL Technology Ltd., Glasgow, Scotland), attached to the anterior midthigh. The use of the *activ*PAL has acceptable validity, practical utility and reliability for the measurement of posture (time spent sitting and lying down) (Davies et al. 2012, Davies, Reilly, and Paton 2012; de Decker et al. 2013). The activPAL monitors were made waterproof with a nitrile sleeve and wrapped in a Tegaderm transparent dressing. During a site visit, trained research personnel applied an additional dressing to attach the monitor to the child's right anterior thigh, positioned halfway between the hip and knee along the midline. Each child was asked to wear the *activ*PAL continuously for at least 72 h and not take it off for any reason, including water activities (like bathing and swimming) and sleeping. Total daily SB was calculated using the ‘sitting time’ as recorded by the *activ*PAL averaged over the valid days (i.e., 24 h of wear) (Kariippanon et al. 2022). Only participants with at least one 24‐h period of wear were included in the final analysis.

##### 2.3.1.2 Screen Time

Daily screen time was measured via the SUNRISE parent questionnaire (Okely et al. 2021). Parents or caregivers were asked to report on how much time their child spent using an electronic screen device such as a smart phone, tablet, video game, watch television or movies, or videos on the internet while they were sitting or lying down, recorded in min/day during the last 7 days (Okely et al. 2021).

##### 2.3.1.3 Restraint

Parents were asked whether their child was restrained for more than 1 h at a time (e.g., in a stroller, car seat or on back of a scooter/moped) using a parent questionnaire developed for SUNRISE (Okely et al. 2021).

##### 2.3.1.4 Compliance With the SB Guidelines

Children were categorized as meeting the screen time, restraint and overall guideline as outlined in Table 1b.

Although the validity and reliability of the screen time and restraint questions are unknown, the use of the SUNRISE parent questionnaire has shown to be practical and culturally appropriate (can be used in both HICs and LMICs), is in line with the WHO 2019 Movement Guidelines for the corresponding age group and has shown to have face validity, for example, in identifying between‐group differences (Abdeta et al. 2024; Hossain et al. 2021).

#### 2.3.2 Parent Demographic Characteristics

Child and parent/caregiver's age, sex, education level and cultural background were recorded. Socioeconomic status (SES) was defined using parent education level.

#### 2.3.3 Gross and Fine Motor Skills

Trained researchers measured the fine and gross motor skill performance of participants using the Ages and Stages Questionnaire‐3 (48 months, ASQ‐3), which is a developmental screening assessment (ASQ‐3 n.d.). The validity and reliability of this questionnaire have been established in children under 5 years (e.g., Fuengfoo et al. 2020; Shariatpanahi et al. 2024).

Participants were asked to perform six tasks for each of fine and gross motor skills. Scoring for the ASQ‐3 involves assigning 10 points for *yes*, 5 points for *sometimes* and 0 points for *not yet* for each task, and from that, a total score for each subscale, gross and fine motor skills, was computed. The total scores for each subscale were the sum of points for all activities performed on fine or gross motor skills and have a maximum score of 60 points. Gross and fine motor skills were categorized as ‘on track’ if total scores were greater than 42.74 and 30.58, respectively; ‘at risk’ if the total score was between 42.74 and 32.78 for gross motor skills and between 30.58 and 15.81 for fine motor skills; and ‘delayed’ if the total score was below 32.78 and 15.81, respectively, in line with the standard ASQ‐3 scoring procedure (ASQ‐3, 2024).

## 3 Data Reduction and Analysis

All statistical analyses were performed using SPSS Statistics for iOS Version 29.0 (IBM Corp, Armonk, NY). Only participants with valid data on at least one of the predictors (screen time, restraint or total daily SB) and outcome (fine or gross motor skills) measures were included for the analysis. Descriptive statistics (mean and standard deviation) were computed for all study variables including total daily SB (h/day) and screen time (h/day). The proportion of children meeting the screen time and restraint guidelines, individually and in combination, was reported. Independent *t* tests and Pearson's chi‐square tests were used to test the difference between sexes.

Univariable regression analysis was used to examine the association between meeting and not meeting the screen time guidelines, restraint guidelines, overall SB guidelines, screen time (h/day) and the total daily SB (h/day) and fine and gross motor skills. A multivariable regression analysis was performed for all variables with a significance of *p* < 0.1 in the univariable analysis.

The association between meeting and not meeting the screen time guidelines, the restraint guidelines, the overall SB guidelines, the screen time (h/day) and total daily SB (h/day) and being on track for fine and gross motor development versus delayed/at risk was examined using univariable logistic regression. Multivariable logistic regression, including sex and SES, was conducted for those variables with a significance of *p* < 0.1 in the univariable analysis. Statistical significance was set at *p* < 0.05.

## 4 Results

### 4.1 Characteristics of Study Participants

A total of 1490 children were recruited; 1394 completed the study protocol and met the data requirements of the present analyses, with 768 from urban and 626 from rural areas. The mean age of participants was 4.4 years (SD = 0.4) with 708 boys and 686 girls. Table 2 shows the descriptive characteristics of participants. Forty‐two per cent (*n* = 567) of children met the overall SB guideline, whereas 50.1% (*n* = 671) and 82.2% (*n* = 1118) of children met the screen time and restraint guidelines, respectively. No statistically significant differences were found between boys and girls in meeting the SB guidelines. For fine motor skills, girls had higher scores than boys (mean ± SD [47.9 ± 13.1] vs. [42.9 ± 16.0] points, respectively, *p* < 0.001). No significant differences in gross motor skill scores were observed between of boys and girls. The percentages of all children who were developmentally on track for fine and gross motor skills were 66.2% and 74.4%, respectively. A higher proportion of girls than boys were developmentally on track (vs. at risk) for fine and gross motor skills.

**TABLE 2:** Descriptive characteristics of the participating children, the overall scores for gross and fine motor skill (mean ± standard deviation) and proportion of children meeting the SB guidelines, stratified by sex.

	Total (*n* = 1394)	Boys (*n* = 708)	Girls (*n* = 686)	*p* value
	Mean (SD)	Mean (SD)	Mean (SD)	
Age	4.4 (0.4)	4.4 (0.4)	4.4 (0.4)	0.473
Total daily SB (hours/day)^a^	7.4 (1.7)	7.5 (1.7)	7.4 (1.6)	0.207
Screen time (hours/day)^b^	1.7 (1.6)	1.7 (1.6)	1.6 (1.5)	0.579
Fine motor skills^c^	**45.4 (15.0)**	**42.9 (16.0)**	**47.9 (13.1)**	**< 0.001**
On track *n* (%)	919 (66.2)	419 (59.5)	500 (73.1)	
Gross motor skills^d^	**50.1 (11.4)**	**50.2 (12.3)**	**51.9 (10.3)**	**0.232**
On track *n* (%)	1037 (74.4)	508 (71.8)	529 (77.1)	
Meeting screen time guideline^e^ *n* (%)	671 (50.1)	341 (50.8)	330 (49.2)	0.912
Meeting restraint guideline^f^ *n* (%)	1118 (82.2)	569 (50.9)	549 (49.1)	0.801
Meeting the overall SB guideline^g^ *n* (%)	567 (42.4)	288 (50.8)	279 (49.2)	0.874

*Note:* Differences between sex tested using independent *t* test. Bold values indicate statistically significant at *p* < 0.05.

^a^ *n* = 881.

^b^ *n* = 1340.

^c^ *n* = 1388.

^d^ *n* = 1394.

^e^ *n* = 1340.

^f^ *n* = 1360.

^g^ *n* = 1338.

### 4.2 Associations Between SB and Gross and Fine Motor Skills Scores

Tables 3 and 4 display the results for the univariable and multivariable regression analysis. In the univariable analysis, meeting the restraint guideline (*p* = 0.016) or overall SB guideline (*p* = 0.007) was favourably associated with fine motor skills. Accumulating 1 h more daily screen time was associated with a 0.55‐point reduction in fine motor skills scores (*p* = 0.027). There was a favourable association between meeting the screen time (*p* = 0.005), restraint (*p* < 0.001) and overall SB guidelines (*p* < 0.001) with gross motor skills scores. Accumulating 1 h more screen time daily was associated with a 0.53‐point reduction in gross motor skill scores (*p* = 0.005). No significant association was observed with total daily SB and fine or gross motor skill scores.

**TABLE 3:** Univariable/multivariable linear regression analysis (corrected for sex and parent education) showing the association between meeting the different components of SB and fine and gross motor skills scores.

	Univariable analysis			Multivariable analysis		
	Fine motor skill			Fine motor skill		
	Unstandardized beta	95% CI	*p* value	Unstandardized beta	95% CI	*p* value
Meeting screen time guideline^a^	1.358	(−0.242, 2.958)	0.096	2.715	(0.667, 4.763)	**0.009**
Meeting restraint guideline^b^	2.547	(0.479, 4.614)	**0.016**	1.36	(−0.222, 2.942)	0.092
Meeting overall SB guideline^c^	2.236	(0.618, 3.854)	**0.007**	2.233	(0.631, 3.835)	**0.006**
Total daily SB (h/day)^d^	0.503	(−0.100, 1.105)	0.102			
Screen time (h/day)^e^	−0.55	(−1.038, −0.062)	**0.027**	−0.031	(−0.110, 0.048)	0.439

	Gross motor skill			Gross motor skill		
	Unstandardized beta	95% CI	*p* value	Unstandardized beta	95% CI	*p* value
Meeting screen time guideline^a^	1.772	(0.545, 2.998)	**0.005**	1.689	(0.462, 2.916)	**0.007**
Meeting restraint guideline	3.052	(1.469, 4.634)	**< 0.001**	3.094	(1.509, 4.679)	**< 0.001**
Meeting overall SB guideline^c^	2.489	(1.250, 3.727)	**< 0.001**	2.405	(1.164, 3.646)	**< 0.001**
Total daily SB (h/day)	0.011	(−0.441, 0.463)	0.962			
Screen time (h/day)^e^	−0.531	(−0.904, −0.158)	**0.005**	−0.504	(−0.877, −0.132)	**0.008**

*Note:* Bold values indicate statistically significant at *p* < 0.05.

^a^ *n* = 1340.

^b^ *n* = 1360.

^c^ *n* = 1338.

^d^ *n* = 881.

^e^ *n* = 1340.

**TABLE 4:** Univariable/multivariable logistic regression (corrected for sex and parent education) showing the association between meeting the different component of SB and odds ratio for being on track for motor skills development (vs. delayed/at risk).

	Univariable analysis				Multivariate analysis			
	On track for fine motor skill (vs. delayed/at risk)		On track for gross motor skill (vs. delayed/at risk)		On track for fine motor skill (vs. delayed/at risk)		On track for gross motor skill (vs. delayed/at risk)	
	OR (95% CI)	*p* value	OR (95% CI)	*p* value	OR (95% CI)	*p* value	OR (95% CI)	*p* value
Meeting screen time guideline^a^	1.278 (1.017, 1.605)	**0.035**	1.439 (1.125, 1.841)	**0.004**	1.286 (1.021, 1.620)	**0.033**	1.419 (1.108, 1.818)	**0.006**
Meeting restraint guideline^b^	1.396 (1.048, 1.860)	**0.023**	1.680 (1.246, 2.266)	**< 0.001**	1.435 (1.073, 1.920)	**0.015**	1.697 (1.256, 2.292)	**< 0.001**
Meeting SB guideline	1.489 (1.178, 1.882)	**< 0.001**	1.653 (1.280, 2.135)	**< 0.001**	1.500 (1.183, 1.903)	**< 0.001**	1.631 (1.261, 2.109)	**< 0.001**
Total daily SB (h/day)^d^	1.034 (0.948, 1.127)	0.453	1.007 (0.918, 1.105)	0.882				
Screen time (h/day)^e^	0.855 (0.790, 0.924)	**< 0.001**	0.911 (0.847, 0.980)	**< 0.001**	0.855 (0.789, 0.926)	**< 0.001**	0.914 (0.849, 0.984)	**0.017**

*Note:* Bold values indicate statistically significant at *p* < 0.05.

^a^ *n* = 1340.

^b^ *n* = 1360.

^c^ *n* = 1338.

^d^ *n* = 881.

^e^ *n* = 1340.

For the multivariable regression analysis, there were favourable associations between meeting the screen time guideline (*p* = 0.009) and overall SB guideline (*p* = 0.006) with fine motor skills scores. Favourable associations were found between meeting the screen time guideline (*p* = 0.007), restraint guideline (*p* < 0.001) and overall SB guideline (*p* < 0.001) and gross motor skills. Accumulating 1 h more screen time daily was associated with a 0.50‐point reduction in gross motor skills scores (*p* = 0.008).

### 4.3 Associations Between SB and the Odds of Being on Track (vs. at Risk/Delayed) for Fine and Gross Motor Skills Development

The univariable logistic regression analysis showed a favourable relationship between meeting the screen time guideline (*p* = 0.035), restraint guideline (*p* = 0.023) and overall SB guideline (*p* < 0.001) and being on track for fine motor skill development. Favourable associations were also found between meeting the screen time guideline (*p* = 0.004), restraint guideline (*p* < 0.001) and overall SB guideline (*p* < 0.001) and being on track with gross motor skills development. Children who accumulated 1 h more screen time per day were less likely to be on track for fine motor skill (*p* < 0.001) and gross motor skill (*p* < 0.001) development. No significant association was observed with the total daily SB and being on track for either fine or gross motor skill development.

For the multivariable logistic regression analysis, favourable associations were found between meeting the screen time guideline (*p* = 0.033), restraint guideline (*p* = 0.015) and overall SB guideline (*p* < 0.001) and being on track for fine motor skill development. There were favourable associations with meeting the screen time guideline (*p* = 0.006), restraint guideline (*p* < 0.001) and overall SB guideline (*p* < 0.001) and being on track for gross motor skill development. Children who accumulated 1 h more screen time were 0.86 times less likely to be on track for fine motor skill development (*p* < 0.001) and 0.91 times less likely to be on track for gross motor skill development (*p* = 0.017).

## 5 Discussion

### 5.1 Findings and Study Implications

Findings from the present study show that accumulating more screen time (h/day) was associated with poorer fine and gross motor skill scores and lowered the odds of being on track for fine and gross motor development. Meeting the screen time guideline and the restraint guideline, individually and in combination, was favourably associated with fine and gross motor skill scores and increased the odds of being on track with fine and gross motor skill development. The present study also showed low adherence to the overall SB guideline (42%), which may indicate that many children between 3 and 4 years are being exposed to excessive screen time and high sedentary time (more than the recommended time per day) and so are more likely to suffer from poorer fine and gross motor development. Findings from the present study however did not show a significant association between total SB and fine and gross motor skills scores.

Similar to findings reported in the present study, a systematic review by Li et al. (2020) and Kwofie, Janssen, and Reilly (2024) reported negative associations between screen time and motor competence. In addition, results reported in a systematic review by Feng et al. (2021) are consistent with those reported in this study, highlighting a positive association between meeting the screen time guideline and motor development in those aged 4–5 years (Feng et al. 2021). The present evidence on total SB is less consistent. The review by dos Santos et al. (2021) reported inconclusive evidence on total SB, with five included studies reporting a negative association, five reporting no association (similar to what has been reported in the current study) and one finding an uncertain association. In addition, Kwofie, Janssen, and Reilly (2024) reported a mix of positive and negative associations between total SB and motor competence.

Findings from this present study add to the existing evidence that limiting certain types of SB (e.g., screen time) is beneficial for children's motor skill development. According to the conceptual model proposed by Stodden et al. (2008), adequate motor competence may promote physical activity and lead to long‐term positive health trajectories. Children with increased screen time may have fewer opportunities to develop gross motor skills, which may increase their adoption of screen usage and other sedentary activities or may decrease their engagement in physical activity (Andrade Neto et al. 2014; Hardy et al. 2018; Stodden et al. 2008).

Although findings from the current study align with previous studies, some differences should be highlighted. Findings from these previous studies did not address the research question addressed in this present study, which examined different components of SB in 3‐ to 4‐year‐olds with a diverse international sample. For example, the reviews by Li et al. (2020) and dos Santos et al. (2021) included children older than 4.99 years of age and did not include any subgroup analysis of SB type or age (Li et al. 2020). In addition, Feng et al. (2021) reviewed adherence to the screen time guideline as the only exposure and only found two studies with preschoolers (aged 3–4.99 years), which may have limited comparability with the present study. These previous reviews also included studies from predominantly HICs, in contrast to the present study.

### 5.2 Strengths and Limitations

The SUNRISE Study has several strengths, including the use of device‐based measures of the total daily SB, and the fine and gross motor skills measures with the ASQ‐3, all of which have been validated within the 3‐ to 4‐year‐old age group and have shown feasibility in global samples. Other strengths are a global and diverse sample of children across different countries, a focus on 3‐ to 4‐year‐olds and different types of SB. The cross‐sectional study design used in this study precludes causal inference and hence further studies (like longitudinal or interventional studies) are required to confirm the direction of association. Screen time was assessed by a parent questionnaire for which the validity and reliability has not yet been established, but associations with outcomes such as gross and fine motor skills in the present study suggest that the questionnaires were measuring something meaningful. The *activ*PAL data had a minimum of 1 day of valid day wear, which may not provide an accurate measure of usual SB. The accelerometer measured SB may also be limited in that it is not be able to distinguish between different types of SB.

## 6 Conclusions

This current study suggests that screen time and restraint are associated with fine and gross motor skill development at age 3–4 years in many countries across a wide range of economic development. It is important that policy makers, health educators and other stakeholders promote and encourage adherence to the SB guidelines.

## Author Contributions

**Nana Kwofie A:** funding acquisition, conceptualization, methodology, writing – original draft, writing – review and editing. **Alex Florindo A:** methodology, validation, data curation. **Amy S HA:** methodology, validation, data curation. **Asmaa El Hamdouchi:** methodology, validation, data curation. **Denise Koh:** methodology, validation, data curation. **Kipling Webster E:** methodology, validation, data curation. **Kar Hau Chong:** methodology, validation, project administration, data curation. **Nyaradzai Munambah:** methodology, validation, data curation. **Penny Cross L:** methodology, validation, project administration, data curation. **Pujitha Wickramasinghe:** methodology, validation, data curation. **Thanh Van Kim:** methodology, validation, data curation. **Dong Hoon Kim:** methodology, validation, data curation. **Anthony Okely D:** methodology, validation, data curation. **Mohammad SorowarHossain:** methodology, validation, data curation. **Mark S.Tremblay:** methodology, validation, data curation. **Hongyan Guan:** methodology, validation, data curation. **Adang Suherman:** methodology, validation, data curation. **Chiaki Tanaka:** methodology, validation, data curation. **Bang N. Pham:** methodology, validation, data curation. **John J. Reilly:** methodology, validation, supervision, data curation, writing – review and editing, conceptualization. **Catherine E. Draper:** methodology, validation, data curation. **Marie Löf:** methodology, validation, data curation. **Amanda E. Staiano:** methodology, validation, data curation. **Hong K. Tang:** methodology, validation, data curation.

## Ethics Statement

The SUNRISE study protocol received ethical approval from the University of Wollongong, Australia (2018/044) and in each country by the applicable ethics committees.

## Conflict of Interest

The authors declare no conflicts of interest.

## Supporting information

Supporting Information

## Data Availability

The datasets supporting the conclusions of this article are available upon request to the SUNRISE team.
