# Supplementary material for: Associations Between Sedentary Behaviour and Fine and Gross Motor Skills in 3‐ to 4‐Year‐Olds: A Secondary Data Analysis From Sunrise International Study Pilot Studies
Source: Child Care Health Dev. 2025 May 12;51(3):e70092. doi: 10.1111/cch.70092 (PMC12069962; doi:10.1111/cch.70092)
Supplement: Supplementary file 1 — Supporting Information [file CCH-51-e70092-s001.docx]

**List of participating countries.**

- Australia
- Bangladesh
- Brazil
- Canada
- China
- Hong Kong
- Indonesia
- Japan
- Malaysia
- Morocco
- Papua
- New Guinea
- Scotland
- South Africa
- South Korea
- Sri Lanka
- Sweden
- USA
- Vietnam
- Zimbabwe
